# Losing the left side of the world: Rightward shift in human spatial attention with sleep onset

**DOI:** 10.1038/srep05092

**Published:** 2014-05-28

**Authors:** Corinne A. Bareham, Tom Manly, Olga V. Pustovaya, Sophie K. Scott, Tristan A. Bekinschtein

**Affiliations:** 1 MRC Cognition and Brain Sciences Unit, 15 Chaucer Road, Cambridge, CB2 7EF, United Kingdom; 2 Department of man and animals physiology, Southern Federal University (SFU), Rostov-on-Don, Russia; 3 Institute of Cognitive Neuroscience, UCL, London, United Kingdom

## Abstract

Unilateral brain damage can lead to a striking deficit in awareness of stimuli on one side of space called Spatial Neglect. Patient studies show that neglect of the left is markedly more persistent than of the right and that its severity increases under states of low alertness. There have been suggestions that this alertness-spatial awareness link may be detectable in the general population. Here, healthy human volunteers performed an auditory spatial localisation task whilst transitioning in and out of sleep. We show, using independent electroencephalographic measures, that normal drowsiness is linked with a remarkable unidirectional tendency to mislocate left-sided stimuli to the right. The effect may form a useful healthy model of neglect and help in understanding why leftward inattention is disproportionately persistent after brain injury. The results also cast light on marked changes in conscious experience before full sleep onset.

At least once almost every 24 hours we undergo a remarkable transformation in which we cease common activities, become relatively unresponsive to external stimuli, and recall little other than fragments of dreams. Whilst probing subjective experience during full sleep is difficult, falling asleep is a process that takes place over several minutes during which people can continue to make responses^1^,^2^. Neuroscientific and cognitive studies of this period indicate that connectivity between frontoparietal and frontotemporal networks diminishes early^3^,^4^,^5^ and, as would be expected given this, high level executive control functions are among the first to decline^1^,^6^.

Our interest was on potential changes in spatial awareness with drowsiness. This had clinical and theoretical origins. Consciousness is commonly separated in two theoretical constructs, wakefulness and awareness^2^,^7^. Sleep onset, a bridge between states in which people may be able to respond to stimuli and yet not integrate all features, such as location^8^, forms an interesting test ground for these relationships relevant to dominant contemporary theories of consciousness^7^,^9^. From a clinical perspective, low alertness in the healthy population may provide a window for investigation of spatial bias that can also result from brain injury. Unilateral spatial neglect is a debilitating condition in which people have difficulties in detecting and localising information from the side of space opposite to a cerebral lesion. Patients may eat food from only one side of the plate or wash and dress only one side of the body. Despite normal vision, they may miss targets in ‘plain view’ on one side of a test page and show marked deviation of the subjective midline^10^. Neglect is common in the acute period following stroke to either brain hemisphere. Left neglect following right hemisphere damage, however, tends to be markedly more persistent than its left hemisphere-right space equivalent^11^. There may be a number of reasons for this including right-hemisphere dominance in aspects of spatial cognition and attention (making it more able to compensate for left hemisphere damage than vice versa^12^) and prolongation of neglect by co-occurring deficits in alertness, themselves associated with right hemisphere function^13^,^14^. The latter argument has been supported by temporary amelioration in rightward bias that follows exposure to stimulating noises, thoughts or medication^15^,^16^,^17^.

There are intriguing suggestions that these alertness dependent lateralised attention shifts echo a pattern detectable in the general population. Following reports that some neurologically normal children with poor sustained attention can also show left neglect^18^,^19^, a series of studies with adults and children have shown significant within-subject rightward shifts in attentional bias with sleep deprivation^20^, reduced night-time alertness^21^, and after long periods of repetitive task performance^22^. In these studies, lowered alertness has been indirectly inferred as a likely consequence of the volunteers' condition (e.g. sleep deprived). Here we examined whether independently indexing alertness using electroencephalography provides a potentially more powerful test of the hypothesised alertness-spatial attention link. Although spatial neglect can be observed across modalities including audition^23^, to date relevant studies with healthy volunteers have used exclusively visual tasks. Because interpretation of behaviour/EEG changes on visual tasks with sleep onset is complicated by blinking and complete or partial eye-closure, here we used an auditory task in which participants kept their eyes closed throughout, facilitating likely drowsiness. Participants were presented with a series of tones at various degrees of divergence from their midline and simply asked to report as quickly as possible whether each had been to their left or right. To support general inference, we were able to index relative alertness during each trial and relate this to the participants' spatial reports on those trials using three different measures.

Sleep onset is marked by suppression of activity in the alpha band, heightened activity in theta and, eventually, the appearance of sleep spindles^24^. These changes are apparent in the continuous EEG, and here, each trial of the task was categorized by a trained clinical electrophysiologist (blind to participants' overt responses) according to an operationalised scale of sleep activity (Hori Scale^24^). In addition, the theta: alpha ratios during the interval leading up to each tone were automatically calculated. This index has previously been used as a sensitive index of sleep onset in previous studies^25^. Finally, sleep onset is linked behaviourally with slowing and greater variability in participants' responses and, ultimately, cessation of responses with full sleep. Examining increased response latency and variability over epochs of task performance therefore provided a third drowsiness measure.

## Results

**Figure 1:**
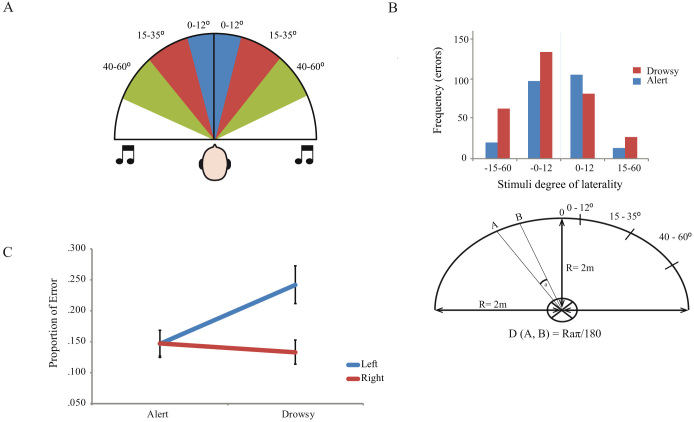
(A.) Identical tones were presented at angles between 0° and 60° from, and equidistant to, the participants' midline. (B.) Error rates on tones within different bands of laterality. Negative degrees are to the left of midpoint, positive are to the right of midpoint. Drowsiness is associated with a marked rise in errors for left tones, including those lateralised as far as 40°–60° to the left. Of note, the tones within 15–60° of laterality are only biased during the drowsy periods – with more left than right errors. (C.) Participants made significantly more left-tone errors than right-tone errors in the drowsy quartile, whilst a similar of left and right tone errors were made in the alert quartile. Error bars represent the standard errors of the means (Alert left: ±.021; Alert right: ±.021; Drowsy left: ±.030; Drowsy right: ±.020).

**Figure 2:**
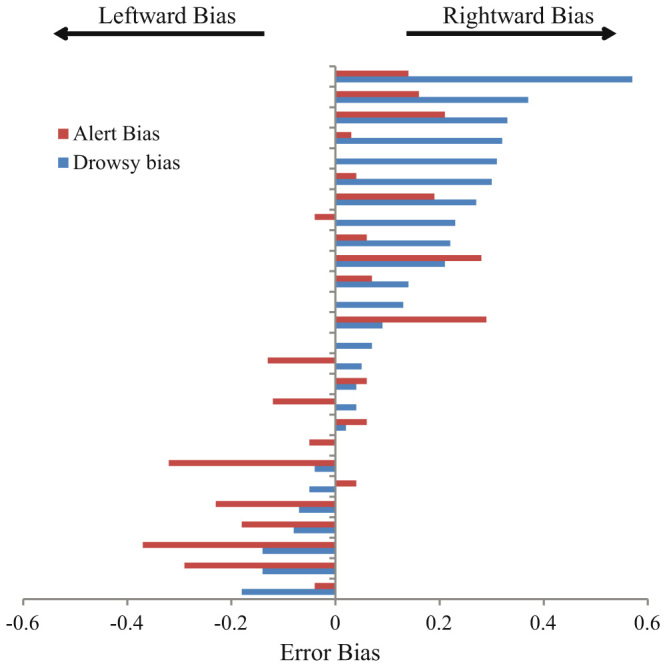
Individual bias scores (proportions left – right tone errors/total errors) during alert and drowsy quartiles. Whether left or right biased initially, almost all the participants (21 of 26) demonstrate a rightward shift in error performance at drowsy periods.

**Figure 3:**
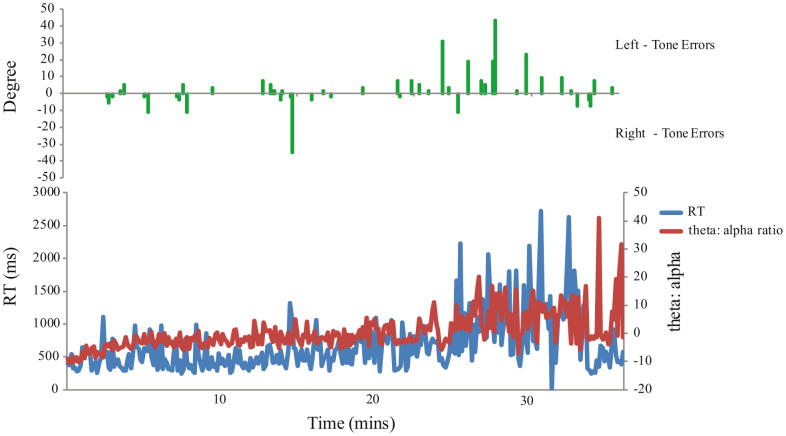
Individual EEG theta: alpha ratio traces, reaction times and error pattern from a single participant across each trial of the session. Early trials are characterised by alertness, relatively fast reaction times and few errors on both left and right tones. By 20-minutes rising drowsiness is following by increased reaction times and a marked increase in errors, almost exclusively on left-tones, including those 15–60° to the left.

To examine the key issue of within-subject changes in performance relatable to alertness levels, EEG lower theta-upper alpha ratios (henceforth ‘theta: alpha ratio’) for the 4 seconds leading up to each tone were automatically calculated and each participants' trials were then divided in quartiles based on this measure. Error rates on the tones (left reported as right and vice versa) were then examined in the upper and lower quartile range (i.e. most drowsy vs. most alert). A striking interaction between arousal (alert, drowsy) and error type (right, left) was observed (Repeated Measures General Linear Model (GLM); *F*(1,25) = 17.53, *p* < 0.001 two-tailed, Cohen's *d* = 1.674). As shown in Figure 1C, a 13.94% (*SD* = 10.49) error rate on left tones during relatively alert performance climbed to 24.67% (*SD* = 15.48) on drowsy trials. In contrast, right-tone error rates slightly *decreased* with drowsiness, from 14.61% (*SD* = 10.53) to 12.92% (*SD* = 10.35). Elevated left-tone error rates during theta: alpha ratio defined drowsy trials were apparent in 21/26 participants (81%; See Figure 2). Odds ratio analyses indicate that a participant was 17 times more likely to show a rightward shift with drowsiness on this task than a leftward shift, or no shift, (*d* = 17.64, *CI* = 4.44 to 70.07). Interestingly, a Repeated Measures ANOVA of only the easier, more lateralised tones (15–60°) showed an interaction between arousal (alert, drowsy) and side of error (left, right) with no difference in errors during alert trials (left *M* = 4.28%, *SD* = 6.93; right *M* = 2.96%, *SD* = 5.65), but more errors on the left (*M* = 16.12%, *SD* = 18.99) than right (*M* = 6.34%, *SD* = 7.98) during the drowsy period; *F*(1, 25) = 7.22, *p* = 0.013 two-tailed, Cohen's *d* = 1.08; See Figure 3 for an example of an individual's performance across time). Interpreting the left and right responses within a Signal Detection Theory framework revealed a statistically significant effect of alertness on sensitivity (*d′* declined from 2.51; *SD* = 1.43 during alert trials to 1.82; *SD* = 1.30 during drowsy trials; *F*(1, 25) = 6.65, *p* = 0.016, Cohen's *d* = 1.03) and criterion ((*c*) increased from 0.03; *SD* = 0.58 during alert trials to 0.49; *SD* = 0.65 during drowsy trials; *F*(1, 25) = 13.42, *p* = 0.001, Cohen's *d* = 1.46) – see supplementary materials for details of these and subsequent analyses.

**Figure 4:**
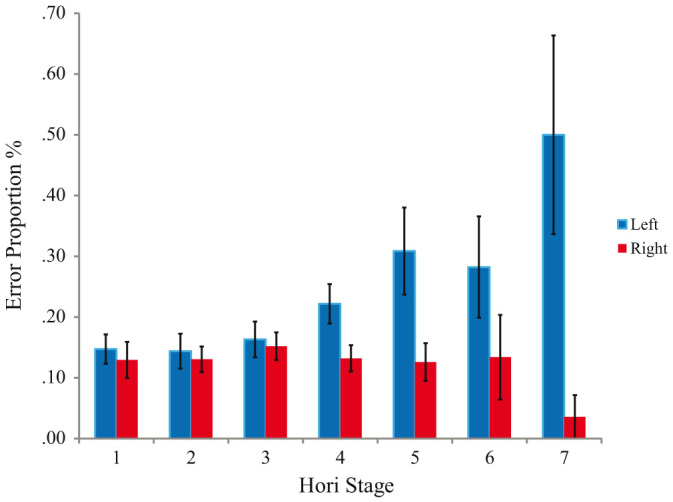
Proportion of left and right errors for trials within each Hori Scale Sleep stage. From Hori Scale 4 left error proportions begin to increase incrementally whilst right error proportions do not show substantial changes with Hori Stage progression.

The same data set was scored by an electrophysiologist (blind to automated theta: alpha ratio, reaction time and accuracy scores) using an adapted version of the Hori Scale^24^. Each 4-second pre-trial interval was categorised as ‘drowsy’ (Hori of 4 or more) or ‘alert’ (less than 4). Although this excluded data from 4 participants who had no trials meeting this definition of drowsy, analysis of error rates on left and right tones according to this distinction on the remainder were entirely consistent with the automated method. There was significant interaction between alertness level (alert, drowsy) and side-of-tone (left, right) on error rates (Repeated Measures GLM; *F*(1,21) = 12.98, *p* = 0.002 two-tailed, Cohen's *d* = 1.57). As before, on left-tones, error rates increased from 15.07% during alert trials (*SD* = 10.59) to 25.26% (*SD* = 17.06) on drowsy trials. For right tones there was again a slight *decrease* in error rates with drowsiness (from 13.15%; *SD* = 8.72 alert to 12.45%; *SD* = 9.95 drowsy; see Figure 4).

Sleep onset is linked with generally increased response latency and also variability^2^; not only might participants' become slower to make judgments, they may prematurely respond to the stimulus before evaluating it, giving a mixture of fast and slow responses. Trials were scored as alert or drowsy based on reaction times (RT see methods). As with the EEG measures, there was a marked interaction between this behaviorally defined alertness (alert, drowsy) and side-of-tone (left, right) in terms of error rates (Repeated Measures GLM; *F*(1,25) = 7.25, *p* = 0.012 two-tailed; Cohen's *d* = 1.08). Left tone error rates increased from 16.75% (*SD* = 15.38) during RT defined alert periods to 23.62% (*SD* = 14.20) during drowsy periods. In the same comparison right-tone errors decreased slightly from 13.88% (*SD* = 10.13) to 12.59% (*SD* = 8.08).

The last frontier of wakefulness transitions is true sleep, behaviourally defined by a prolonged failure to respond to stimuli^2^. To examine our results independent of EEG defined alertness, each participant's behavioural responses were divided into sub blocks of 10-trials and the number of omissions in each successive block counted. The relative proportion of left and right tone errors in blocks with 2 or more omissions was then compared with those for the same participants in which no omissions occurred. Nineteen participants met the criteria for admission into the ANOVA of having data from blocks in which no omissions occurred and from blocks in which 2 or more omissions occurred. Again, a striking interaction between tone laterality (left, right) and alertness (alert, drowsy) was observed with drowsiness leading to a substantial increase in left-tone errors (Repeated measures GLM; *F*(1, 18) = 9.2404, *p* = 0.008 two-tailed Cohen's *d* = 1.42). The rate of errors on left-tones increased from 18.60% (*SD* = 12.41) in blocks with no omissions to 22.67% (*SD* = 15.09) in blocks with two or more omissions. In contrast – and consistent with the EEG analysis – error rates on right-tones *decreased* from 14.86% (*SD* = 8.63) to 8.44% (*SD* = 11.74) in the same comparison. The degree to which participants evidenced a disproportionate increase in errors on left-tones with drowsiness (based on the theta: alpha ratio analysis reported above) was significantly correlated with level of drowsiness as indexed by Hori scores, RT index and number of trials with no responses, indicative of sleep onset. As would be expected from their different derivations and properties, convergence between the 4 indices of alertness was significant but not absolute (see supplementary materials for analyses).

## Discussion

The results show that reduced alertness, indexed by EEG or behaviour, in healthy participants is associated with a markedly asymmetric increase in error rates in lateralising left- compared with right-located auditory stimuli. Whilst it is possible that this reflects an alertness-related change in specifically auditory processes there are no known precedents for this in the literature. In contrast, the result is strongly consistent with a raft of studies in clinical and healthy populations that have shown changes in performance on visual tasks linked with reduced alertness, that have been attributed to changes in spatial attention. The current study extends this literature by, for the first time using direct EEG measurement as well as behavioural alertness indices and demonstrating that, like the biases of spatial neglect, that analogous patterns can be detected in the auditory as well as visual modalities.

Lateralised spatial awareness has been conceptualised as a competition between the cerebral hemispheres; the left hemisphere pushing attention to the right and vice versa. Such competition produces a finely balanced system vulnerable to perturbation. Unbalanced competition in which the intact hemisphere comes to dominate its impaired rival helps in understanding why spatial neglect is so ubiquitous following unilateral brain injury from stroke. Even in health, it has been argued that a process that differentially activates one or other hemisphere (such as language for the left hemisphere) can induce bias towards contralateral space^12^. On this basis, any non-uniform effect of reduced alertness/sleep on the hemispheres could produce detectable shifts in spatial bias. Such effects have indeed been suggested by studies showing differential cessation rates in right and left hand tapping during sleep onset^26^. One possibility is that suppression of a postulated right hemisphere dominant ‘cerebral activation’ network^27^, necessary for transition to sleep, may lead to the rightward bias we observed.

Another account is that reduced cognitive control affects the integration of characteristics of a stimulus, including its location, and that this unmasks an underlying rightward pull in normal human spatial attention of currently unknown origin. This would be consistent with both the normal and clinical literature on lateralised shifts with low alertness and distraction^15^,^16^,^17^,^20^,^21^,^22^,^28^ and studies showing disengagement of prefrontal and parietal regions^3^,^4^,^5^ with consequent reduction in cognitive control and target detection^29^. The magnitude and reliability of the rightward shift reported here suggests a tractable method for examining this directionally specific effect, including its relationship with hemispheric specialisation in other functions such as language.

Damage to the right hemisphere has long been reported to cause disproportionate impairment in the maintenance of an alert state. The results here suggest that patients with these lesions may be doubly compromised, the rightward bias of unbalanced interhemispheric competition being exacerbated by prolonged drowsiness, now clearly linked with normal rightward bias. Whilst a variety of spatial interventions have been used in the rehabilitation of the disorder, the results here confirm that the maintenance of alertness should be another important therapeutic target.

## Methods

Ethics was obtained from the Cambridge Psychological Research Ethics Committee (CPREC) prior to all testing. All experimental protocol, including EEG methods, was approved by CPREC. All methods were carried out in accordance with the approved guidelines. Informed consent was obtained from all participants prior to testing.

### Participants and stimuli

Twenty-six healthy volunteers (mean age 24.15, SD = 5 all right-handed, 17 women) were individually tested in a quiet, dark room in a comfortable reclined chair. They were presented with harmonic complex tones that differed only in their location, falling in an arc between 0° and 60° of the midline, balanced for angle and side (see Figure 1A). The tones were recorded in free field using in-ear microphones. Original recordings plus stereo flipped duplicates formed the stimuli set such that there were no non-spatial cues to location (see supplementary methods). Presentation was weighted such that tones within 1.86–12° were presented six times each (N = 72), tones 15–35° were also presented six times each (N = 72), tones 40–60° were presented once (N = 12) and finally midpoint tones were presented three times each (N = 6). Stimuli were presented randomly, with a different randomisation for each participant. Participants were asked to report whether it was to the left or right by pushing one of two buttons left- and right-located on a response box resting centrally on the abdomen. To control for any effect of lateralised movements, 9 participants were randomly assigned to respond using the right and left thumbs, 9 with the index and middle finger of the left hand, and 8 with the index and middle finger of the right hand. In each case the left button/leftmost digit was used to indicate the ‘left’ response.

### Procedure

After providing informed written consent, participants were fitted with the EGI electrolyte 129-channel cap (EGI systems). Stimuli were presented via in-ear headphones. Each trial began with a random interval of between 5–8 seconds before the tone. Participants were asked to keep their eyes closed throughout the task and to respond as quickly and as accurately as possible. If no response was detected the experiment would proceed to the next trial after 5 seconds. No feedback was given on accuracy. Participants were invited to relax and not worry if they fell asleep, as they would be woken by incremental increases in the volume of the stimuli (which occurred after three successive failures to respond). Overall the task lasted approximately 40–50 minutes. At the end of the task, the participant was sat upright and the EEG cap was removed. The data was acquired using a Mac computer using Netstation software.

### Indices of alertness

#### Automated EEG analysis

Previous research indicates that sleep-onset EEG changes tend not to be evenly distributed across alpha and theta bands and that fine-grained analysis may enhance measurement sensitivity. For theta, the 4-5 Hz band and for alpha the 10-11 Hz or have received empirical support^30^. This is consistent with our own preliminary studies of sleep-onset during cognitive task performance that identified 4-6 Hz and 10-12 Hz bands as most sensitive. Accordingly these parameters were applied to the current study. These theta: alpha sub-band ratios were computed from the 129 electrodes over the 4 secs preceding each tone. Each trial for each participant was then categorized as relatively drowsy (top 25% of theta: alpha ratio scores) or alert (lowest 25%). It should be noted that this relative within-subject method will categorise an equal number of trials as drowsy and alert regardless of absolute levels (i.e. a participant who was notionally ‘alert’ throughout would still have 25% of trials tagged as ‘drowsy’). *Visual EEG analysis*: EEG traces for all participants throughout the task were examined by a trained clinical electrophysiologist who was blind to the automated theta: alpha ratio scores, RTs and participants' judgments. The 4 seconds preceding stimulus onset in each trial was given a score on Hori Scale (1–9). For dichotomous analysis, as is conventional, scores 1–3 were categorised as alert trials and stages 4 and above as drowsy. The alert (wake) stages were identified by the presence of alpha (H1-2), with relaxed wakefulness indicated by alpha suppression (H3), and drowsiness determined by alpha flattening (H4), and deeper sleep determined by the presence of spindles (H9)^24^. *Reaction time analysis*: Reaction times can vary according to the difficulty of the stimulus categorization on a trial as well as variations in alertness. To characterise periods of slow and variable responding with minimal confound of task difficulty, a moving average RT was calculated for each for each trial based on its own and the previous 9 trials' RTs. Where non-responses occurred, the moving average began again 10 trials later to ensure each value was based on a representative (in terms of difficulty) number of sequential trials. For each participant, each trial was then categorized according to whether this value fell above a grand mean of rolling averages for that participant. The size of a standard deviation (a measure of RT variability across trials) is generally related to the absolute magnitude of the mean from which it is derived (slow responses vary more than fast ones). The coefficient of variation (CV; standard deviation/mean) forms an index of variability that is relatively independent of the mean^31^. Accordingly, as for the RTs, a rolling CV was calculated based on the current, and preceding 9 trials', RTs. This was then categorized as falling above or below each participant's grand average CV. For the combined RT and CV analysis, a trial was categorized as ‘alert’ if both values fell below the respective means for that participant and ‘drowsy’ if both were above. Trials with mixed outcomes were therefore excluded from the analysis. As with the theta: alpha ratio analysis, this relative within-subject measure categorized trials as ‘alert’ and ‘drowsy’ even in participants who were potentially subjectively ‘alert’ (or ‘drowsy’) throughout the task.

## Author Contributions

C.A.B., T.M., S.K.S. and T.A.B. designed the research; C.A.B. and T.A.B. performed the research; C.A.B., T.M., O.V.P. and T.A.B. analyzed the data; and C.A.B., T.M. and T.A.B. wrote the manuscript and supplementary information.

## Supplementary Material

Supplementary Information Supplementary Information
