# Supplementary material for: Losing the left side of the world: Rightward shift in human spatial attention with sleep onset
Source: Sci Rep. 2014 May 28;4:5092. doi: 10.1038/srep05092 (PMC4035582; doi:10.1038/srep05092)
Supplement: Supplementary Information [file srep05092-s1.doc]

Losing the left side of the world: Rightward shift in human spatial attention with sleep onset

Corinne A. Bareham¹, Tom Manly¹, Olga V. Pustovaya^1,2^, Sophie K. Scott^3^, Tristan A. Bekinschtein¹

¹ MRC Cognition and Brain Sciences Unit, 15 Chaucer Road, Cambridge, CB2 7EF, United Kingdom

^2^ Department of man and animals physiology, Southern Federal University (SFU), Rostov-on-Don, Russia.

^3^ Institute of Cognitive Neuroscience, UCL, London, United Kingdom

Running Title: Sleep onset and spatial attention

Corresponding author: Bareham, C. A.

Address: MRC Cognition and Brain Sciences Unit, 15 Chaucer Road, Cambridge, CB2 7EF, UK.

Telephone: 01223 355294

**Supplementary materials**

# Supplementary Methods

## Stimuli Construction

The aim was to develop a set of stimuli that were identical save for cues as to their spatial location relative to the listener and which were relaxing and pleasant to facilitate drowsiness. Accordingly, a D guitar chord (wider bandwidth sounds are easier to localise than single tones) was recorded once (lowest frequency 0Hz, highest 5000 Hz based around 146.382 Hz, duration 551ms). This chord was then played via a single loudspeaker at a fixed volume at set points within a 180° semicircle. These examples were recorded via in-ear microphones worn by an experimenter seated in the centre of the semicircle. The loudspeaker was positioned at a height of 1m - approximately ear-level of the seated listener. The stimulus was played at equivalent increments starting from 270° left of the listener’s position to 90° to the right. Tones were recorded at a 2m radius with the degree of spatial laterality equated from the distance around the arc of the semicircle using the formula:

*D (A, B) = Raπ/180*

Where *R* is the length of the radius and *a* represents the angle of the distance between the two points on the arc of a circle’s circumference. Nineteen of the stimuli were recorded from 270/90° positions approaching midpoint in 4° increments (approximately 14 cm apart). The remaining six increments narrowed to approximately 2° (approximately 6.5cms) starting from 346/12° positions. These smaller increments within 12° either side of midpoint were thought to allow for a finer measure of spatial laterality judgments around participants’ midpoint. Finally, two midpoint stimuli were included (one as approached from the right and one from the left), making a total of 52 stimuli; 25 left-sided, 25 right-sided and two midpoint stimuli. The 52 stimuli recorded on both the right and left side were then stereo-flipped using Audition© software. Stereo-flipping ensured that a stimulus recorded from the left was directly mirrored at the exact same spatial position on the right (and vice versa) and made sure that any extraneous noise was counterbalanced across stimuli. Thus a total of 104 sound file stimuli were created, each 400ms in length. Piloting indicated a ceiling effect on the left/right categorisation of tones > 60° in either direction. Accordingly only tones ≤ 60° were used in the main experiment. Piloting showed no difference in the accuracy with which the laterality of left and right stimuli were judged *t*(3) = 2.18, *p* = 0.12.

## Procedure

Participants were reclined in a comfortable chair into a horizontal position positioned in a dark electrically isolated, and sound-proofed room. A pillow and blanket were available to participants if they felt it would facilitate drowsiness. Before beginning the task, participants were tested on the stimuli to ensure the volume was adequate and that they could distinguish accurately between the left and right tones – with tones at various levels of difficulty presented. No participant had current hearing impairments or any history of injury/condition affecting hearing acuity. Once the experimenter was confident that the participant could accurately distinguish between the left and right tones, the task would commence. Participants were instructed to keep their eyes closed throughout the task, to respond on every trial with a left/right response even if they were unsure, and to relax and not worry if they fell asleep.

## Pre-processing of EEG Data

# EEG was sampled at a rate of 250Hz. No filtering took place during data acquisition. Off-line the EEG data were then filtered at 0.5 – 40Hz band pass and segmented into epochs comprising the 4 seconds preceding each tone. Files were then exported to MATLAB for remaining pre-processing. Data were re-referenced to the average of 129 channels. Bad channels were rejected based on visual inspection and interpolated using spherical spline interpolation of the voltages from the remaining electrodes^32^. No more than 5 channels were interpolated per participant^33^ with affected segments rejected if more than 5 channels were noisy. Bad segments due to muscle movements or eye blinks (rare due to eyes-closed performance) were rejected manually by eye. An automatic rejection method was run to aid identification of those epochs to be considered for rejection. This automatic rejection method identified epochs by extreme values of beyond -150µv and beyond 150µv. Following the exclusion of the first 10 trials of each session as practice, the remaining clean trials were used in the analyses. The mean number of trials removed due to EEG artefact was 63.00 (19.49%, *SD* = 51.94, 15.98%) leaving a mean of 259. 39 (*SD* = 50.73) trials per participant for the analysis.

# Supplementary Results

## Performance in lateralising stimuli during periods of relatively high and low alertness as defined by the upper and lower quartiles of each participants’ theta:alpha ratios.

The first analysis reported in the main paper is an interaction between error rates for left- and right-located stimuli and alertness as defined by the upper and lower quartile range of each participants’ theta:alpha distribution (*F*(1,25) = 17.53, *p* < 0.001, Cohen’s *d* = 1.674). The results in detail were as follows. There was a statistically significant effect of alertness (alert error rate = 14.03%, *SD* = 6.00, drowsy error rate = 18.43%, *SD* = 8.80; *F*(1,25) = 5.53. *p* = 0.027). There was no overall effect of side-of-stimulus (error rate on left trials = 19.06%, *SD =* 10.63, right trials = 13.71%, *SD =* 9.39; *F*(1,25) = 2.95, *p* = 0.099). Kolmogorov-Smirnov tests on the error rate data showed adequate levels of normality (*p* > 0.01 and in most cases > 0.05). Measures of skew and kurtosis were also consistent with normal distinctions (skew <1 >-1 and kurtoses <3 >-3).

### Signal Detection Theory Analysis

To analyse the results within a Signal Detection Theory (SDT) framework *d’* (sensitivity) was calculated using **ϕ^-1^***(H)* - **ϕ^-1^***(F)* and *c* (criterion) using (**ϕ^-1^***(H)* + **ϕ^-1^***(F)*)/2 where *H* was the proportion of left-stimulus trials with a correct response and *F* was the proportion of right-stimulus trials with a left response. The *d’* and *c* values were entered into separate repeated measures ANOVA with the within-subject factors of alertness (trials from the lower quartile of each participant’s theta:alpha distribution vs. trials from the upper quartile). There was a statistically significant effect of alertness on sensitivity (*d’* declined from 2.51; *SD =* 1.43 during alert trials to 1.82; *SD* = 1.30 during drowsy trials; *F*(1,25) = 6.65, *p* = 0.016, Cohen’s *d* 1.03) and criterion (*c*) increased from 0.03; *SD =* 0.58 during alert trials to 0.49; *SD =* 0.65 during drowsy trials; *F*(1,25) = 13.42, *p* = 0.001, Cohen’s *d* = 1.46). It is important to note however that the more ‘relaxed’ criterion associated with drowsy trials reflects the increased rate with which participants reported left-stimuli as having occurred on the right. We cannot infer from the current results whether this was because they were more likely to judge ‘right’ when uncertain about a stimulus when relatively drowsy or actually experienced these stimuli as occuring on the right.

### Effect of theta:alpha ratio defined alertness on report accuracy for the most lateralised tones

The second result in the main paper re-ran the repeated measures ANOVA on error rates with the within-subject factors of theta: alpha defined alertness and side-of-stimulus using only trials with the most lateralised tones (15º - 60º). The results in detail were as follows. There was a statistically significant main effect of alertness with error rates on these trials increasing from 3.61% (*SD =* 4.77) on relatively alert trials to 10.70% (*SD* = 10.76) on relatively drowsy trials (*F*(1,25) = 11.45. *p* = 0.002, Cohen’s *d* = 1.35). There was also a statistically significant effect of side-of-stimulus with error rates being greater overall for left (10.01%; *SD =* 10.23) than right tones (4.33%; *SD =* 5.64; *F*(1,25) = 5.94, *p* = 0.022, Cohen’s *d =* 0.98). The alertness x side-of-tone interaction (*F*(1, 25) = 7.22, *p =* 0.013; Cohen’s *d* = 1.08) was reported in the main paper. In summary, whilst errors on these ‘easy’ trials were relatively infrequent, when they did occur it was almost invariably on left-stimulus trials during periods of relative drowsiness.

### The effect of response hand/s and sex.

All participants had self-reported right-hand preference but were allocated at random to respond with their left, right or both hands. There was no significant group differences in overall average reaction time (*F*(2,23) = 1.087, *p* = 0.354) or omission rates (*F*(2,23) = 1.063, *p* = 0.362). The repeated measures ANOVA on error rates as a function of theta: alpha defined alertness and side-of stimulus reported above (using stimuli at all degrees of lateralisation) was repeated with hand-of-response as a between-subjects factor. There was a statistically significant interaction between alertness and hand-of-response (*F*(2,23) = 4.13, *p* = 0.03) reflecting differential changes in error rates between alert and drowsy trials among the three groups (right-hand responders alert trial error rate 14.96%; *SD =* 4.27; drowsy trial error rate 22.52%; *SD =* 6.18; left-hand responders alert 13.41%; *SD* = 7.09; drowsy 20.97%; *SD* = 9.63; both-hand responders alert 13.88%; *SD* = 6.63; drowsy 11.18%; *SD =* 5.57). No response-hand group showed an overall differential pattern of errors for left- and right-stimulus trials (side-of-stimulus x hand-of-response interaction *F*(2,23) = 0.78, *p* = 0.47) and, critically, there was no 3-way interaction between hand-of-response, alertness and side-of-stimulus (*F*(2,23) = 0.81, *p* = 0.46). In short, whilst the group sizes are relatively small (they were included to balance rather than specifically investigate hand-of-response) the main finding of a markedly disproportionate increase in errors on left-tones with drowsiness was not driven primarily by one group.

Seventeen women and 9 men took part in the study. Sex was included as a between-subjects factor in the repeated measures ANOVA on error rates with the within-subjects factors of theta: alpha defined alertness and side-of-stimulus. No significant interactions were observed (sex x alertness *F*(1,24) = 0.004, *p* = 0.95; sex x side-of-stimulus *F*(1,24) = 1.19, *p =* 0.29; sex x alertness x side-of-stimulus *F*(1,24) = 2.24, *p* = 0.15). For brevity, the effects of hand-of-response and sex, which were largely negligible, are included at the end of the other ANOVA analyses reported below.

## Performance in lateralising stimuli during periods of relatively high and low alertness as defined by Hori ratings.

The main paper reports a significant interaction between alertness as defined by electrophysiologist’s Hori rating scores from the EEG and side-of-stimulus (*F*(1,21) = 12.98, *p =* 0.002 two-tailed, Cohen’s *d* = 1.57). In detail, a repeated measures ANOVA was conducted with the within-subject factor of Hori-defined alertness (high vs. low) and side-of-stimulus (left vs. right). This revealed statistically significant effect of alertness with error rates climbing from 14.09% (*SD =* 5.98*)* during alert trials to 19.30% (*SD =* 11.64) during drowsy trials (*F*(1,21) = 6.03, *p =* 0.023). There was no overall effect of side-of-stimulus (*F*(1,21) = 3.09, *p* = 0.093). Note data from 4 participants were excluded due to insufficient trials being categorised as >3. When hand-of-response and sex were separately entered as a between-subjects factors no significant interactions with alertness or side-of-stimulus, or 3-way interactions, were observed (hand-of-response x alertness *F*(2,19) = 0.04, *p* = 0.96; hand-of-response x side-of-stimulus *F*(2,19) = 0.80, *p* = 0.46; hand-of-response x alertness x side-of-stimulus *F*(2,19) = 0.18, p = 0.83; sex x alertness *F*(1,20) = 0.05, *p* = 0.83; sex x side-of-stimulus *F*(1,20) = 2.05, *p* = 0.17; sex x alertness x side-of-stimulus *F*(1,20) = 1.45, *p =* 0.24).

The main Hori-defined alertness analysis was repeated using SDT parameters. Repeated measures ANOVA with the within-subject factor of Hori-defined alertness and the dependent variable *d’* (calculated as above) revealed a non-significant change in *d’* from 2.02 (*SD =* 0.92) during Hori-defined alert trials to 1.83 (*SD* = 1.31) during Hori-defined drowsy trials (*F*(1,20) = 2.34, *p* = 0.14, Cohen’s *d =* 0.80). The same analysis on *c* (again, as defined above) indicated a statistically significant change from alert (0.084, *SD =* 0.46) to drowsy trials (0.48, *SD =* 0.63; *F*(1,20) = 9.81, *p =* 0.005, Cohen’s *d* = 1.40).

## Performance in lateralising stimuli during periods of relatively high and low alertness as defined by reaction time speed and variability.

The main paper reported an interaction between alertness, as defined by an index based on RT speed and variability, and side-of-stimulus. In detail, a repeated measures ANOVA was conducted on error rates with the within-subjects factors of alertness (high vs. low RT index) and side-of-stimulus (left vs. right). This revealed no overall statistically significant effect of alertness on errors (alert trial error rate 15.13%, *SD =* 7.47; drowsy trials 18.52%, *SD =* 7.49; *F*(1,25) = 2.88, *p* = 0.10) and a trend towards an overall effect of side-of-stimulus (error rate on left trials 20.76%, *SD =* 13.62; right trials 12.77%; *SD* = 7.18; *F*(1,25) = 3.86, *p* = 0.061) as well as the reported significant RT defined alertness x side-of-stimulus interaction (*F*(1,25) = 7.25, *p =* 0.012; Cohen’s *d* = 1.08). Neither hand-of-response nor sex interacted with the main factors or interactions on separate repeated measures ANOVA analyses (hand-of-response x alertness *F*(2,23) = 0.82, *p* = 0.45; hand-of-response x side-of-stimulus *F*(2,23) = 0.27, *p =* 0.77; hand-of-response x alertness x side *F*(2,23) = 0.47, *p* = 0.63; sex x alertness *F*(1,24) = 2.63, *p =* 0.12; sex x side-of-stimulus *F*(1,24) = 0.83, *p* = 0.37; sex x alertness x side-of-stimulus *F*(1,24) = 0.42, *p =* 0.52).

The main RT-defined alertness analysis was repeated using SDT parameters. Repeated measures ANOVA with the within-subject factor of RT-defined alertness and the dependent variable *d’* (calculated as above) revealed a statistically significant change in *d’* from 2.87 (*SD =* 1.29) during RT defined alert trials to 1.66 (*SD* = 0.86) during RT defined drowsy trials (*F*(1,25) = 16.81, *p* < 0.001, Cohen’s *d =* 1.64). The same analysis on *c* (again, as defined above) indicated a statistically significant change from alert (-0.08, *SD =* 0.89) to drowsy trials (0.45, *SD =* 0.38; *F*(1,25) = 12.96, *p =* 0.001, Cohen’s *d* = 1.44).

## Performance in lateralising stimuli during periods of relatively high and low alertness as defined by absent responses.

The final result presented in the main paper was an interaction between alertness (as defined by task periods marked by missed responses vs. no missed responses) and side-of-stimulus for the 19 participants with eligible data. In detail, a repeated measures ANOVA was conducted on error rates with the within-subject factors of omission defined alertness (alert vs. drowsy) and side-of-stimulus (left vs. right). This revealed no statistically significant main effect of alertness (*F*(1,18) = 0.57, *p* = 0.46) and a trend towards a significant effect of side-of-stimulus (left-tone error rates 19.23%; *SD =* 11.62; right-tone 13.29%; *SD =* 7.37; *F*(1,18) = 4.32, *p* = 0.050) in addition to the alertness x side-of-stimulus interaction (*F*(1, 18) = 9.2404, *p =* 0.008 two-tailed Cohen’s *d* = 1.42). Neither hand-of-response nor sex interacted with the main factors or interactions on separate repeated measures ANOVA analyses (hand-of-response x alertness *F*(2,16) = 0.16, *p* = 0.86; hand-of-response x side-of-stimulus *F*(2,16) = 1.43, *p =* 0.27; hand-of-response x alertness x side *F*(2,16) = 0.007, *p* = 0.99; sex x alertness *F*(1,17) = 0.05, *p =* 0.83; sex x side-of-stimulus *F*(1,17) = 0.43, *p* = 0.52; sex x alertness x side-of-stimulus *F*(1,17) = 0.34, *p =* 0.56). Of the participants included in this analysis, 7 responded with their right hand, 8 with the left hand, and 4 with the thumbs of both hands.

The main omission-defined alertness analysis was repeated using SDT parameters. Repeated measures ANOVA with the within-subject factor of omission-defined alertness and the dependent variable *d’* (calculated as above) revealed a non-statistically significant change in *d’* from 2.38 (*SD =* 0.83) during RT defined alert trials to 1.96 (*SD* = 1.70) during RT defined drowsy trials (*F*(1,18) = 0.65, *p* = 0.43). The same analysis on *c* (again, as defined above) indicated a statistically significant change from alert (0.03, *SD =* 0.51) to drowsy trials (1.25, *SD* 0.0.82; *F*(1,18) = 39.19, *p <* 0.001, Cohen’s *d* 2.95) in those 11 participants will eligible data.

# Are individual differences in the degree to which performance changed with relative drowsiness related to how drowsy participants became?

As reported, 21 of the 26 participants showed a pattern of increased errors on left-tones with drowsiness. An issue is whether the degree of change in error rates is related to how drowsy the participants became. To examine this, correlations between changes in the SDT measure *c* between theta: alpha ratio defined alert and drowsy periods (drowsy *c –* alert *c*) and each of the indices of alertness previously reported were examined. The *c* parameter is useful in taking into account the relative increase in errors to left- and right-tone trials in a single index (see above). Change in *c* were significantly correlated with the mean of all theta: alpha ratios (Pearson’s *r*(26) = 0.49, *p =* 0.01), the maximum Hori score achieved (Pearson’s *r*(26) = 0.44, *p =* 0.03), the number of trails categorised as drowsy according to the combined RT slowness and variability metric (Pearson’s *r*(26) = 0.45, *p =* 0.02) and, strikingly so, the number of trials with no response (indicative of sleep onset; Pearson’s *r*(26) = 0.89, *p* <0.001). Theta:alpha ratio scores had a mean of 0.16 (*SD =* 0.16, range -50.12 – 57.97 and were normally distributed (Kolmogorov-Smirnov> 0.05, skew <1 >-1, kurtoses <3 >-3). Raw Hori scores had a mean of 2.89 (*SD =* 0.98 range 1.19 – 4.44) and were normally distributed Kolmogorov-Smirnov > 0.05) although, as would be expected in participants generally sufficiently awake to continue making responses, were skewed towards lower values (Skew -0.2, Kurtosis -1.08). Approximately equal numbers of trials were categorized as Hori 1, 2, 3 and 4 (mean percentage of trials categorized as Hori 1 = 21.85%, *SD =* 26.70, Hori 2 = 21.31%, *SD* = 16.27, Hori 3 = 18.74%, *SD* = 12.18, Hori 4 = 26.83%, *SD* = 26.47, Hori 5 = 9.00%, *SD =* 13.24, Hori 6 = 1.16% *SD =* 2.04 with the remaining categories having fewer than 1% of trials respectively).

# Convergence between the indices of alertness.

The different indices of alertness used here have rather different properties. The automated theta: alpha upper and lower quartile split, for example, will create equal numbers of trials in each category regardless of the absolute level of alertness of the participant. In contrast, the Hori ratings rely on a more absolute categorisation of the EEG data but are, of course, prone to some variations in human rater performance and criteria. The RT index split was based on the mean values of a rolling RT average and coefficient of variation, both of which would equally divided all trials for each participant but the definition relied on convergence between these indices (rolling RT and coefficient of variation both below respective means, or both above), which would not necessarily produce equal numbers. The omissions analysis was between blocks of 10 trials with more than 2 omissions (the number of which varied between participants including several with none) and blocks with no omissions.

Convergence was first examined in a series of repeated measures ANOVAS with the within-subject factor of alertness (alert vs. drowsy as defined by the relevant index) and the dependent variables of the other continuous indices of alertness. The results, presented in Supp. Table 1 show that, regardless of how alertness was categorized, the other continuous variables showed marked differences in the hypothesised direction with strong effect sizes. For example, reaction times were slower and there were significantly more missing responses (suggestive of sleep onset) in trials automatically categorised as drowsy/alert according to EEG theta: alpha ratio or by independent Hori ratings. Similarly, theta: alpha scores and Hori ratings were significantly higher during drowsy trials as defined by the RT index or omissions. The one slight exception to this general pattern was the difference in theta: alpha ratio scores between trials categorised as alert/drowsy according to Hori scores of 1 vs. >3, which only reached marginal significance. It is important to note, however, that a relatively small proportion of trials (and for some participants, none) were categorized as drowsy according to Hori ratings.

Supp. Table 1.

|  | | Dependent variable | | | |
| --- | --- | --- | --- | --- | --- |
| Alert/drowsy categorisation variable | | theta: alpha ratio *M (SD)* | Hori scale *M (SD)* | Reaction time *M (SD)* | Omissions *M (SD)* |
| Theta:alpha ratio | Alert |  | *2.30 (0.98)* | *1150.51ms (515.31)* | *2.12 (3.64)* |
|  | Drowsy |  | *3.51 (1.42)* | *1477.32ms (561.63)* | *8.89 (10.66)* |
| *p,*  Cohen’s *d* | |  | 0.001, 1.49 | 0.001, 1.61 | 0.005, 1.24 |
| Hori scale  (1 vs. >3) | Alert | *2.34 (3.10)* |  | *1198.37ms (526.94)* | *5.73 (7.37)* |
|  | Drowsy | *4.03 (14.80)* |  | *1496.19ms (526.04)* | *12.46 (16.65)* |
| *p,*  Cohen’s *d* | | 0.06, 0.82 |  | <0.001, 2.69 | 0.04, 0.93 |
| RT index (RT + variability) | Alert | *-1.56 (2.36)* | *2.63 (1.02)* |  | *0.0 (0.0)* |
|  | Drowsy | *1.28 (2.88)* | *3.13 (1.06)* |  | *18.61 (20.56)* |
| *p,*  Cohen’s *d* | | 0.005, 1.23 | 0.005, 1.23 |  | <0.001, 1.85 |
| Omissions | Alert | *-1.36 (1.74)* | *2.48 (0.97)* | *1311.79ms (430.67)* |  |
|  | Drowsy | *3.38 (5.82)* | *3.37 (1.21)* | *1738.63ms (498.09)* |  |
| *p,*  Cohen’s *d* | | 0.007, 1.44 | <0.001, 2.06 | <0.001, 2.98 |  |

*Supp. Table 1.* Results of repeated measures ANOVAs with the within-subject factor of alertness category (alert vs. drowsy according to the relevant classifier) and the listed dependent variables.

Secondly we conducted a series of pairwise Chi-squared analyses in which the number of trials in which categorization converged/diverged for each index was first calculated for each participant and then summed across the group. The results showed associations between the categories in the expected directions with small (theta: alpha – RT, Hori-RT), medium (theta: alpha – omissions, Hori-omissions), and large (theta: alpha – Hori, RT-omissions) effect sizes.

Supp. Table 2.

|  |  | Hori | |  |  |  |
| --- | --- | --- | --- | --- | --- | --- |
|  |  | Alert | Drowsy |  |  |  |
| Theta:alpha | Alert | 1337 | 327 | 1664 | Chi-square Yates 589.54 *p* < 0.0001; Pearson 591.26, *p* < 0.0001. Cohen’s *d* equivalent 0.93 | |
|  | Drowsy | 645 | 1011 | 1656 |  |  |
|  |  | 1982 | 1338 | 3320 |  |  |
|  |  | RT | |  |  |  |
|  |  | Alert | Drowsy |  | Chi-square Yates 80.23 *p* < 0.0001; Pearson 81.03, *p* < 0.0001 Cohen’s *d* equivalent 0.38 | |
| Theta:alpha | Alert | 455 | 638 | 1093 |  |  |
|  | Drowsy | 293 | 924 | 1217 |  |  |
|  |  | 748 | 1562 | 2310 |  |  |
|  |  | Omissions | |  |  |  |
|  |  | Alert | Drowsy |  |  |  |
| Theta:alpha | Alert | 1319 | 172 | 1491 | Chi-square Yates 151.15 *p*< 0.0001; Pearson 152.28, *p* < 0.0001 Cohen’s *d* equivalent 0.47 | |
|  | Drowsy | 1001 | 429 | 1430 |  |  |
|  |  | 2320 | 601 | 2921 |  |  |
|  |  | RT | |  |  |  |
|  |  | Alert | Drowsy |  |  |  |
| Hori | Alert | 1142 | 1614 | 2756 | Chi-square Yates 98.84, *p* <0.0001; Pearson 99.47, *p* <0.0001 Cohen’s *d* equivalent 0.30 | |
|  | Drowsy | 494 | 1334 | 1828 |  |  |
|  |  | 1636 | 2948 | 4584 |  |  |
|  |  | Omissions | |  |  |  |
|  |  | Alert | Drowsy |  |  |  |
| Hori | Alert | 3207 | 432 | 3639 | Chi-square Yates 314.78 *p* < 0.0001; Pearson 316.01, *p* < 0.0001 Cohen’s *d* equivalent 0.48 | |
|  | Drowsy | 1530 | 677 | 2207 |  |  |
|  |  | 4737 | 1109 | 5846 |  |  |
|  |  | Omissions | |  |  |  |
|  |  | Alert | Drowsy |  |  |  |
| RT | Alert | 1584 | 12 | 1596 | Chi-square Yates 990.12 *p* < 0.0001; Pearson 992.40, *p* < 0.0001 Cohen’s *d* equivalent 1.16 | |
|  | Drowsy | 1223 | 1076 | 2299 |  |  |
|  |  | 2807 | 1088 | 3895 |  |  |

*Supp. Table 2.* Chi-square 2x2 tables of agreement between the 4 indices of alertness in determining trials as alert or drowsy.

**Supplementary References**

32.Junghöfer, M., Elbert, T., Tucker, D. M., & Rockstroh, B. (2000). Statistical control of artifacts in dense array EEG/MEG studies. *Psychophysiology*, *37*(4), 523-532.

33. Jacoby, O., Hall, S. E., & Mattingley, J. B. (2012). A crossmodal crossover: Opposite effects of visual and auditory perceptual load on steady-state evoked potentials to irrelevant visual stimuli. *NeuroImage*, *61*(4), 1050-1058.
